# Objective Methods of Monitoring Usage of Orthotic Devices for the Extremities: A Systematic Review

**DOI:** 10.3390/s23177420

**Published:** 2023-08-25

**Authors:** Devi Baruni Devanand, Angela E. Kedgley

**Affiliations:** Department of Bioengineering, Imperial College London, London SW7 2AZ, UK; d.devanand19@imperial.ac.uk

**Keywords:** orthosis, brace, splint, compliance monitoring, adherence, objective, temperature sensor, pressure sensor, accelerometer, step counter

## Abstract

Orthoses are commonly prescribed to relieve symptoms for musculoskeletal and neurological conditions; however, patients stop wearing orthoses as recommended for many reasons. When considering the effectiveness of orthoses, there needs to be an objective way to monitor whether participants wear the orthosis as instructed, because if this is not followed, the orthoses will not work as intended. This review aimed to identify, summarise, and compare objective methods used to measure compliance with orthoses applied to the extremities. Databases (Scopus, Web of Science, Embase, CINAHL, and MEDLINE) were searched for eligible studies. Twenty-three studies were accepted in the final review, including five studies that employed upper limb orthoses, two that employed hip orthoses, and fifteen that employed lower limb orthoses. To measure compliance objectively, studies utilised temperature sensors, pressure sensors, accelerometers, a step counter, or a combination of sensors. All sensor types have their own advantages and disadvantages and should be chosen based on study-specific parameters. Sensor-derived monitoring provides quantitative, objective data that are beneficial in both clinical and research settings. The ideal solution to monitoring compliance would consist of both objective and user-reported aspects that, in combination, would provide an all-encompassing picture of the orthotic treatment prescribed.

## 1. Introduction

Orthoses are medical devices that are prescribed to control joint alignment, correct deformity, or prevent injury. They are usually a non-invasive and inexpensive treatment option. They are commonly used to aid patients with a variety of conditions, such as osteoarthritis [1], rheumatoid arthritis [2], chronic unilateral stroke [3], cerebral palsy [4], spastic hemiplegia [5], and shoulder injury prevention [6].

Compliance is defined as “the extent to which the patient’s behaviour matches the prescriber’s advice” [7] and is a crucial factor in determining whether or not a treatment is working. Information about patient compliance has benefits in clinical and research settings. Knowledge regarding patient compliance with recommendations for orthosis use provides clinicians with information about patients’ behaviour towards orthoses and offers the ability to intervene and change the course of their use. This could include recalling and improving fit, providing further education about use, stopping the use of the orthotic device, or switching to another treatment option. Ultimately, understanding patient behaviour through treatment compliance can help patients progress in their treatments by enabling clinicians to provide better care. Moreover, orthoses are often prescribed to relieve symptoms of conditions [1]; however, the evidence highlighting the effectiveness of these orthoses is underdeveloped [8,9]. Complicating this, when research is conducted to evaluate orthosis effectiveness, there needs to be an objective way to monitor whether participants wear the orthosis as instructed, because if guidelines are not followed, the effectiveness of orthosis use as prescribed cannot be assessed. Monitoring compliance is also necessary to understand the efficacy and impact of orthotic treatment on overall health and wellbeing [10]. In the UK alone, it is reported that there are at least 1.2 million orthotics users [11], leading to an expenditure amounting to at least GBP 48 million annually during the years 2015–2016 [12]. With many stakeholders and a huge weight on the economy, particularly in publicly funded healthcare settings, it is imperative to know whether orthoses are effective, and to what extent compliance influences this [13].

There are many reasons why patients do not adhere to orthotic treatment. For example, previous studies have shown that patients stopped wearing hand orthoses as recommended due to both discomfort and the inability to perform activities of daily living [14]. Discomfort can be experienced by patients both physically, such as due to an ill-fitting orthosis, skin irritation, or sleep disturbance [14,15,16], and psychologically, such as the feeling of being different from peers and having the perception of a social stigma associated with wearing a brace [17]. Furthermore, it has been reported that psychosocial factors, such as low self-esteem, impacted compliance with treatment [17]. Treatment duration has also been shown to affect compliance [17]. Equally important are the patient’s attitude towards the treatment and their confidence in the clinicians providing it; both have been shown to impact compliance [17]. 

Patient compliance with orthoses is usually monitored using patient diaries [18], questionnaires [19], or interviews [20]. However, these records can be subjective, and patients have been found to overestimate their levels of compliance [16,21]. Furthermore, data from patient-reported compliance-monitoring methods are prone to social desirability bias, with patients over-reporting ‘good behaviour’ [22]. These methods are also vulnerable to recall bias, as wear time is easily forgotten [14]—especially when compliance is assessed weeks or months after prescription. Equally important to note is that the very fact that compliance is measured can influence patient behaviour [23]. Consequently, there is a need to monitor patient compliance with orthoses in an objective way. In this review, an objective monitoring method was defined as one that did not require user intervention to record wear time and was not influenced by the wearer, caregiver, or prescriber’s beliefs. 

Objective compliance monitoring has been explored thoroughly in spinal orthoses, and several techniques, such as the use of strap tension to determine wear time [24], a load-monitoring system [25], and most commonly temperature sensors [26,27,28], have been used to monitor adherence to orthosis treatment. The use of objective compliance monitoring in spinal orthosis is well evidenced [29]. Yet, in comparison, there is a lack of evidence regarding objectively monitoring compliance in orthoses applied to the upper and lower limbs. 

Previous systematic reviews have investigated compliance of patients wearing orthotics and have summarised methods of assessing adherence to guidelines for orthoses use [16,17]. However, the vast majority of these reviews discuss patient-reported compliance monitoring; there are no reviews exclusively summarising compliance-monitoring methods for orthoses that are objective. Therefore, the aim of this review is to identify, summarise, and compare objective methods used to measure compliance with orthoses applied to the extremities.

## 2. Materials and Methods

### 2.1. Literature Search

**Figure 1:**
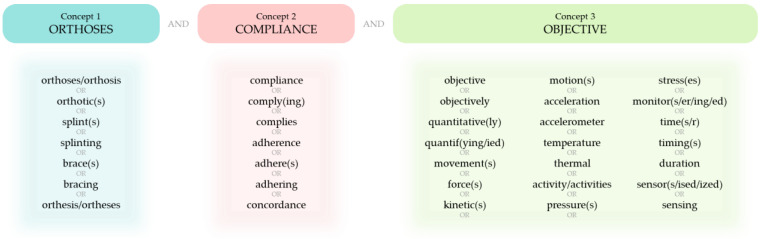
The search strategy developed by generating and combining a list of terms synonymous with the key concepts: orthosis, compliance, and objective.

**Figure 2:**
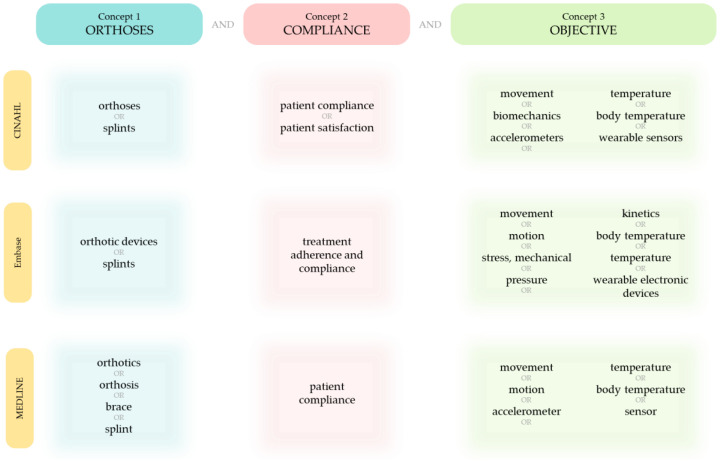
The list of MeSH terms developed for each database (CINAHL, Embase, and MEDLINE) using the main concepts.

A systematic literature search following the PRISMA guidelines [30] was performed to find eligible publications from inception to March 2023. The aim of the literature search was to find studies that have used objective methods to monitor user compliance with orthotic devices for the extremities—namely, in the upper limb, lower limb, and the hip. A search strategy was developed by generating and combining a list of terms synonymous with the key concepts: orthosis, compliance, and objective (Figure 1). Specifically, the strategy combined the related terms for each key concept using the OR operator and merged the key concepts together using the AND operator. The strategy was then used to search titles and abstracts of publications on the electronic databases Scopus, Web of Science, Excerpta Medica dataBASE (Embase), the Cumulative Index to Nursing and Allied Health Literature database (CINAHL), and MEDLINE. Additionally, in order to also retrieve relevant articles by topic, Medical Subject Headings (MeSH) were used alongside the strategy when searching the health science databases Embase, CINAHL, and MEDLINE. As the classification of MeSH terms differs between the databases, a list of MeSH terms was developed for each database by using the main concepts to check for relevant subject headings in that database (Figure 2). When executing the search, the MeSH terms for each key concept were combined with the search terms of the respective concept using the OR operator and, like before, the key concepts were then merged using the AND operator.

### 2.2. Eligibility Criteria

Publications, limited to human studies, were included if the abstract described the use of any objective method of monitoring splint compliance. Studies were excluded if they were case reports, conference proceedings, written in a language other than English, review articles, or registrations from clinical trials. Eligible studies could utilise any study design, such as randomised controlled trials. There were no restrictions on the type of participants, as the emphasis was on the monitoring technique. Publications from all over the world were examined, and participants could be of any age, gender, and ethnicity. All eligible studies were required to investigate compliance with an orthotic intervention only, and publications were excluded if the study investigated the compliance with orthoses that were not worn on the extremities. Any type of compliance-monitoring technique was acceptable so long as the technique was objective, that is, not reliant on an individual—user, caregiver, or clinician—to report the outcome. This, therefore, excludes techniques such as patient diaries, but includes sensor-based or real-time compliance-monitoring methods.

### 2.3. Screening and Selection 

Once duplicate records were excluded, the titles and abstracts of the remaining articles were screened to identify studies that fit the eligibility criteria. Furthermore, orthoses in the included studies were required to be removable by the participant or caregiver, non-invasive, and used for treatment of the upper limb, lower limb, or the hip.

### 2.4. Data Extraction

Data regarding the orthosis type, the medical condition for which the orthosis was used, the study design, sample size, type of participants, participant age range, participant gender, the country in which the study was carried out, the compliance-monitoring technique, the instructions for orthosis use, and the pros and cons of the monitoring techniques were extracted.

### 2.5. Data Analysis

A PRISMA flow chart documenting the selection process was compiled. Compliance-monitoring techniques were compared and the pros and cons for each were tabulated.

## 3. Results

### 3.1. Literature Search

**Figure 3:**
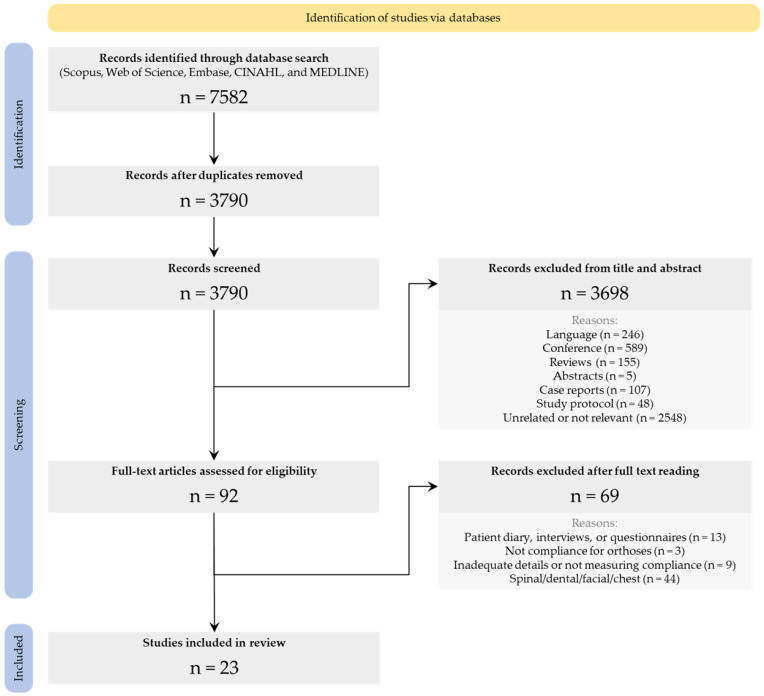
The flow of information and the article selection process through the different phases of the systematic review, following the characteristic steps outlined in the PRISMA diagram [30].

The search returned 1141 records from Scopus, 1166 records from Web of Science, 2435 records from Embase, 845 records from CINAHL, and 1995 records from MEDLINE. Combined, 7582 records were obtained from the five databases, and once duplicates were removed, 3790 records remained. These records were then screened based on their title and abstract, resulting in 3698 articles being excluded as they did not fit the inclusion criteria. Reasons for exclusion included articles not being published in English, conference proceedings, case reports, abstracts only, registrations for clinical trials, and review articles (Figure 3). Furthermore, records were excluded if they were irrelevant, that is, not about usage of orthoses. The remaining 92 records required full-text screening and, following full-text review, 69 studies were excluded as they did not include objective compliance-monitoring methods, did not provide enough detail regarding how compliance was monitored, did not monitor compliance with orthoses, or the orthoses were not applied to the extremities (Figure 3). The remaining 23 studies were accepted in the final review. All 23 studies included in the review used objective compliance-monitoring methods to evaluate participants’ use of their orthoses.

### 3.2. Study Demographics

The 23 studies that were included in the review included three randomised controlled trials [31,32,33] and 20 observational studies [21,34,35,36,37,38,39,40,41,42,43,44,45,46,47,48,49,50,51,52] (Table 1). Seven studies collected data from healthy participants [34,35,38,39,42,43,44,45], fifteen studies recruited patients [21,31,32,33,36,37,41,46,47,48,49,50,51,52], and one study included data from both healthy and patient participants [40]. The sample size of the included studies ranged from two to 124. Five studies used upper limb orthoses [21,34,39,45,46], two used hip orthoses [33,47], and sixteen used lower limb orthoses [31,32,35,36,37,38,40,41,42,43,44,48,49,50,51,52]. The included studies were conducted in a variety of geographical regions. The majority were conducted in Europe—five in the United Kingdom [32,36,37,42,44], three in the Netherlands [34,40,41], three in Germany [35,38,48], two in Switzerland [21,46], and one in Portugal [39]. Out of the remaining nine studies, seven were conducted in the United States of America [31,45,47,49,50,51,52], one in China [33], and one in Australia [43]. Studies employed orthoses used for a wide range of medical conditions, including diabetes [32,40,41,42,48,52], clubfoot [31,37,49,50], postoperative care after shoulder surgery [21,45,46], carpel tunnel syndrome [39], hip fracture prevention [33], hip dysplasia [47], lower limb fracture recovery [35,51], foot drop [44], and spinal cord injury [36]. Three studies did not mention a specific condition but investigated upper limb orthoses for the impairment of the shoulder, arm or hands [34], foot and lower limb disorders [43], and calf muscle unloading [38]. 

### 3.3. Compliance Monitoring

Out of the twenty-three studies that were included in the review, fourteen studies utilised temperature sensors to monitor compliance [21,33,34,39,40,41,42,43,45,46,47,48,49,50], six studies used pressure sensors [31,32,35,39,51,52], four studies used step counters [36,40,41,44], and six studies used accelerometers [33,37,38,42,43,44]. Sixteen studies used one type of sensor in their methodology to monitor compliance [21,31,32,34,35,36,37,38,45,46,47,48,49,50,51,52], while seven used a combination of sensors: three studies used temperature sensors alongside accelerometers [33,42,43], two studies used temperature sensors and step counters [40,41], one study used temperature sensors with pressure sensors [39], and one study utilised accelerometers alongside step counters [44]. Where patients were participating, duration of orthosis use ranged from a week [37,40,41] to just under two years [49], and they were advised to either wear the orthosis all the time [21,31,33,38,49,51] or during ‘nights and naps’ [47,50]. The sampling rate of the data collected from the sensors ranged from reading 50 times a second (50 Hz) [35] to once every 45 min [50].

### 3.4. Temperature Sensors

Temperature sensors were used in 14 studies and were embedded in orthoses applied to upper limbs [21,34,39,45,46], hips [33,47], and lower limbs [40,41,42,43,48,49,50]. Eight studies used temperature sensors alone to monitor compliance [21,34,45,46,47,48,49,50] (Table 2), while six studies used temperature sensors alongside other sensors: three studies used temperature sensors alongside accelerometers [33,42,43], two studies alongside step counters [40,41], and one with pressure sensors [39]. The temperature sensors used in the studies were found to accurately measure wear time [33,34,39,45,46,47] and, in some cases, were able to obtain readings without direct sensor–skin contact [34]. One study found that temperature-based sensors were more than 99% accurate at monitoring compliance when compared to orthosis wear time recorded by volunteers [45]. Using a wear time estimation algorithm or a cut-off threshold, the temperature sensor data were used to discern donning and doffing of the orthosis [21,33,34,40,42,43,45,46,47,48,50]. A trained algorithm was found to estimate wear time from unseen temperature data with mean wear time errors ranging from 0.5 to 8.3% at the arm and 0.13 to 13% at the chest [34]. Overestimation in self-reported wear time was clear in two studies when collecting self-reported compliance data alongside the use of the temperature sensor [21,33]. Nine studies collected data from patient populations, demonstrating the usability of this method in a clinical setting [21,33,40,41,46,47,48,49,50]. A range of sampling rates were used in the studies—the most frequent reading was taken every 30 s [33], three studies sampled temperature readings every minute [34,40,41,43], one study recorded readings every three minutes [42], five studies every 15 min [21,45,46,48,49], and one every 45 min [50]. A limitation of a lower sampling rate is that it could be possible miss the times when the orthosis was donned or doffed, leading to less accurate estimations of wear time [40,45]. While two studies reported concerns that patients knowing about the temperature sensor monitoring compliance could affect results [45,49], the use of temperature sensors to monitor compliance was found to improve long-term adherence and facilitate the early detection of non-compliance [48]. Furthermore, one study highlighted the low cost of temperature sensors [39], and the small size of the temperature sensors used in these studies means that they have the potential to be used in other orthoses for both upper and lower limbs [40].

Despite the many positive observations, concerns about the use of temperature sensors were raised. In some studies, wear time was determined in part by evaluating ambient temperature [33,40,42,43], and although ambient temperature was found not to affect sensor readings in one study [43], one of the main reported drawbacks of using temperature sensors to monitor compliance is that readings are likely to be affected by the local climate [40]. One study collected data during the winter and, therefore, the algorithm was not trained to monitor compliance on warmer days, which leads to the possibility of less accurate wear time estimations when temperatures are higher [48]. Moreover, wear time could also be estimated incorrectly, as users may leave the sensor-embedded orthosis in a warmer environment [45]. Additionally, sensors with less contact with the body were found to overestimate compliance [45]; therefore, factors such as the thickness of clothing could affect sensor accuracy [47]. Other reported limitations of this method were a positive correlation between the amount of data stored on the sensor and the error in wear time estimation [34], and some required the modification of orthoses to integrate the sensor [43].

**Table 2:** Details of the studies using temperature sensors alone to monitor compliance, ordered by year published.

Author and Year	Orthosis Prescribed	Study Aim(s)	Population Demographics	Description of Technology	Instructions for Use	Wear Time Estimation	Pros of Method	Cons of Method
Sangiorgio et al., 2016 [50]	Mitchell Ponseti brace sandals for clubfoot	Assess difference between prescribed and measured brace use, difference between parent-reported use and measured brace use, extent to which brace compliance affects risk of relapse.	48 patients (37 male and 11 female) aged 6 months to 4 years	Two wireless temperature loggers (SmartButton, ACR Systems, Surrey, BC, Canada) consisting of programmable data acquisition, a 3 V battery, and data storage capacity of 2048 readings for data collection every 90 min by each sensor, offset by 45 min, for 4 months. Sensors attached to outside of braces and above the heel.	Wear brace during night and naps.	Data imported to MATLAB and data from both sensors were synchronised to make a single dataset for each patient. A baseline temperature was established using the mean temperature of the dataset, and wear time was determined by finding data points above the baseline.	Objectively measured compliance. Beneficial to clinicians when interpreting parental reports of brace use.	Sampling rate was limited.
Ehrmann et al., 2018 [48]	Insoles for diabetes	Determine when patients become nonadherent to diabetic footwear. Observe possible effects of gender on adherence.	26 patients (18 male and 8 female) aged 59–76 years	Temperature sensor (Orthotimer, Rollerwerk Medical Engineering & Consulting, Balingen, Germany) was embedded into the longitudinal arch of one insole. Temperature was measured within footwear every 15 min. Sensors stored data for 100 days before overwriting the oldest data.	-	An optimal cutoff temperature of 25 °C was found by testing the sensor in healthy participants. Temperatures higher than cutoff temperature were classified as worn and lower temperatures as not worn.	Monitored compliance objectively. Improved long-term adherence and early detection of non-compliance. Patients could not feel the sensor.	No information regarding patient activity and mobility. Temperatures in footwear could have exceeded 25 °C due to environmental factors and not just wear.
Grubhofer et al., 2019 [21]	Abduction shoulder brace for post-arthroscopic rotator cuff repair	Analyse abduction brace wearing behaviour in patients who underwent arthroscopic rotator cuff surgery.	50 patients (23 male and 27 female) aged 28–79 years	Temperature sensor (Orthotimer, Rollerwerk Medical Engineering, Balingen, Germany) was invisibly placed in abdominal belt of brace. Sensor recorded surrounding temperature every 15 min.	23 h/day wear time for 6 weeks postoperatively.	If the measured temperature was above 35 °C, it was recorded as wear time. Data from the sensors were read out using computer software and displayed wear time for each day since sensor activation.	Clear to see overestimation in self-reported wearing time, so useful to have invisible sensor.	Self-reported wear time was done by patient estimation in outpatient visit; questionnaires would have been better to compare with temperature sensor. Software only shows hours worn per day and not when during the day.
Richards et al., 2020 [49]	Abduction brace for clubfoot	Observe daily orthosis wear time in patients successfully treated with the Ponseti method. Determine compliance of patient caretaker with prescribed brace treatment.	124 patients (83 male and 41 female) aged less than 3 months	Temperature sensor (iButton, Maxim Integrated Products, San Jose, CA, USA) embedded in shoe recorded temperature every 15 min. Held up to 3 months of data.	Prescribed 22 h/day for the first 90 days, then 12 h/night until 2 years old.	-	Highlighted true usage of braces.	Awareness of foot temperature being measured could have influenced brace wear. Few sensors failed to record data during a time interval.
Sood et al., 2021 [45]	Shoulder sling for postoperative use	Investigate accuracy of temperature sensors positioned in shoulder slings. Assess whether sensor could discern difference between body temperature and hot environment.	4 healthy participants (3 male and 1 female) aged 25–32 years	Compact (3.35 × 5.64 × 1.8 cm; 12.75 g) data loggers (Onset HOBO MX2201, Onset Computer Corporation, Bourne, MA, USA) with internal microprocessor, data storage, and sensors to measure contact temperature. Data was sampled every 15 min and transferred via Bluetooth. Sensors were in three locations within the sling: inner region of the bolster touching the abdomen, medial elbow region, and palmar surface of the carpometacarpal joint.	Wear the sling as much as possible but free to remove the sling to perform daily activities.	An algorithm was used to estimate wear time. Start of a wear period was determined by a temperature increase. Temperature had to remain above a threshold value for at least 30 min and not exceed a maximum threshold value. End of a wear period was categorised by a fall in temperature and the temperature being lower than a threshold value for 30 min.	Accurately measured compliance (>99% accuracy). Algorithm could discern temperature difference when donned/doffed.	Data recorded at 15 min intervals, so could underestimate wear time. Sensors with less body contact overestimated compliance. User could “cheat” by leaving sling in a warm environment. Hawthorne effect could alter patient behaviour if they are aware they are being monitored.
Swarup et al., 2021 [47]	Abduction brace for residual acetabular dysplasia	Validate efficacy of part-time bracing. Determine relationship between brace wear time and correction of pathology.	26 patients around 6 months of age	Temperature sensor (iButton, Maxim Integrated Products, San Jose, USA), costing USD 75, placed in the posterior thigh region of the abduction brace.	Wear brace for nights/naps and return in 6 months for follow-up.	Temperature higher that 75 °F was defined as the orthosis being worn and less than or equal to 75 °F was defined was not worn.	Used differences in body and ambient temperatures, and wear patterns to determine temperature thresholds.	Inaccuracies could arise from temperature sensor depending on whether the brace was worn over or under clothing.
Grubhofer et al., 2022 [46]	Abduction shoulder brace for post-arthroscopic rotator cuff repair	Investigate whether compliance with immobilisation influences healing. Define compliance rate associated with tendon-repair post rotator cuff repair.	50 patients (23 male and 27 female) aged 28–79 years	Temperature sensor (Orthotimer, Rollerwerk Medical Engineering, Balingen, Germany) was invisibly placed in abdominal belt of brace. Sensor recorded surrounding temperature every 15 min.	23 h/day wear time for 6 weeks postoperatively.	If the measured temperature was above 35 °C, it was recorded as wear time. Data from the sensors was read out using computer software and displayed wear time for each day since sensor activation.	Monitored compliance objectively.	Patient not informed about sensor until after 6 weeks—could affect behaviour.
Haarman et al., 2022 [34]	Upper limb orthoses for impairment of the shoulder, arm, or hands	Validate method to estimate orthosis wear time using temperature sensors attached to the upper body. Assess if two temperature sensors are better than one to estimate wear time. Investigate the effect of sampling time on wear time estimation.	15 healthy participants (7 male and 8 female) aged 24–67 years	Miniature (diameter: 17 mm, height: 6 mm) data loggers (DS1922L Thermochron iButtons, Maxim Integrated Products, San Jose, USA) that measure and store temperature. Attached to the body using elastic straps positioned around the chest and forearm. Data were sampled at 1 min. Android smartphone application cued user to don or doff.	Remove and re-attach straps at specified time-points. Sixteen hours of non-use and eight hours of donning and doffing as instructed by the smartphone app (intervals between cues ranged from 15–60 min).	Data obtained from temperature sensors were used to train decision tree classification algorithm to estimate wear time.	Accurate wear time estimation without direct sensor–skin contact. Accurate estimation during donning and doffing. Algorithm was evaluated with unseen data to minimise bias.	As sampling time increased, the data stored increased, and wear time error and estimation error range increased. Potential discrepancy between actual and reported timestamp due to reaction time of user (on smartphone app). Data not collected on warm days, so not trained for high temperatures.

### 3.5. Pressure Sensors

Pressure sensors were used to monitor compliance in six studies looking into upper [39] and lower limb [31,32,35,51,52] orthosis use. Five studies used pressure sensors alone to monitor compliance [31,32,35,51,52] (Table 3), while one study used pressure sensors alongside temperature sensors [39]. As was the case with temperature sensors, the use of pressure sensors alongside patient diaries highlighted overestimation in self-reported compliance in some cases [31,52], but it was also found that the presence of the sensor did not influence the reported rates of wear [31]. One study looking into pressure sensor fitted insoles found that the system had a mean bias of 11.58 N, and the limits of agreement were ±125 N [35]. These sensors were reported to provide data representative of patient behaviour that could be collected continuously in out-of-clinic settings [51] and for extended periods; in one case, as long as three months [52]. A disadvantage of this monitoring system, however, is that it could be difficult to apply the same technology to other devices, even if applied to the same anatomical region [31]. Furthermore, in one study, the monitoring system could be removed by the user [52], meaning that a user could still wear their orthosis and the system would not be able to accurately deduce if they were wearing it. Other issues reported included problems engaging with associated technology, such as a smartwatch that alerted the user when they were applying too much pressure to a particular region in their feet [32], and in one study, there were no data collected when the orthosis was not worn [52].

**Table 3:** Details of the studies using pressure sensors alone to monitor compliance, ordered by year published.

Author and Year	Orthosis Prescribed	Study Aim (s)	Population Demographics	Description of Technology	Instructions for Use	Wear Time Estimation	Pros of Method	Cons of Method
Morgenstein et al., 2015 [31]	Denis-Brown bar and shoes for clubfoot	Investigate brace wear rates. Determine if a sensor influenced wear rates.	67 patients (47 male and 20 female) aged 0–3 years	1.5 inch-square pressure sensor (Interlink Electronics, Camarillo, CA, USA) attached to sole of shoe and connected to data logger box containing circuit board by wires. Data logger collected voltage readings from the pressure sensor that were then exported to Microsoft Excel for processing.	Wear the device for 24 h per day for 3 months (except when bathing).	Baseline voltage readings collected for 10 min while participant wore the orthosis. Baseline data were used to set a threshold value using an algorithm. At all data points above the threshold, brace was considered to be worn.	Clear to see overestimation in self-reported compliance. Presence of sensor did not influence the reported rates of wear.	Does not address different types of braces used for clubfoot.
Döbele et al., 2016 [35]	Walking boot for lower extremity fracture recovery	Assess and evaluate validity and reliability of activity-monitoring device for real-time feedback and long-term measurement of partial weight bearing.	20 healthy participants under the age of 50 years	Insole sensor system consisting of 15 sensors (13 pressure sensors, a temperature sensor, and a measuring acceleration. Only pressure sensors were used to obtain data for this study. Included embedded battery power supply. Data collected recorded on data chip and downloaded wirelessly (ANT Technology). Sample rate of 50 Hz.	Wear walking boot instrumented with insole and complete course of 500 m containing stairs with partial weight bearing (15 kg).	MATLAB script used to identify steps taken and analysed maximum force of every step.	User did not have to operate insole themselves. Data recording was automatically activated as soon as sole was in motion and entered standby mode when not in motion. No problems encountered when fitting into orthotic boot. Wireless data read out was reliable. Maintained accuracy regardless of load applied.	Data were not recorded for extended periods of time (a week and more), so full potential of device was not explored.
Najafi et al., 2017 [52]	Footwear for diabetes	Observe adherence to alert-based offloading with a pressure-sensitive insole system.	12 patients aged 52–71 years	Smart insole system (SurroSense Rx system, Orpyx Medical Technologies Inc., Calgary, AB, Canada) consisting of two pressure-sensing insoles and a smartwatch. Pressure data collected from the plantar surface of foot through insoles transmitted to smartwatch. Each insole contained eight pressure sensors. Provided alerts when safe thresholds for pressure were exceeded.	Wear device for 3 months.	Adherence was defined as the time when footwear with the insole and smartwatch were worn together. Pressure data were obtained from the smartwatch.	Objectively monitored daily adherence during a long period of time (3 months). Self-reported adherence was higher than recorded by sensors. Technology was accepted by patients.	System can be removed by patient. Cannot tell if patient was not wearing prescribed footwear, wearing inserts with no watch, or if they removed insert from footwear. No data collected when device was not worn.
Abbott et al., 2019 [32]	Insoles for diabetes	Investigate effectiveness of active insole system in preventing diabetic foot ulcer recurrence.	58 patients (51 male and 7 female) aged 50–76 years	Plantar pressure measuring insole system (SurroSense Rx, Orpyx Medical Technologies, Calgary, Canada) weighing 45 g, consisting of 0.6 mm flexible, pressure-sensing inserts connected to a smartwatch using ANT+ wireless communication protocol. Each insert contained eight pressure sensors located at plantar surface of foot. Sampling rate of 8 Hz. Smartwatch provided alerts encouraging patients to offload and had a battery life of 2 days (350 mAh rechargeable). Battery life of sensing inserts was 1 week (80 mAh rechargeable).	Wear footwear throughout day-to-day life.	Compliance data obtained when insole was worn inside shoes were connected to smartwatch. For every minute of wear, readings from the previous 15 min were categorised as high, medium, and low pressure.	Measured cumulative pressure applied over time. Intervention was self-directed. Provided high-pressure alerts to allow patients to take action and offload.	Calculation of wear time did not include times when patient wore shoes with insoles without being connected to the smartwatch or when shoes were worn without insoles. Low perceived aesthetic value and problems engaging with smartwatch technology reported as reasons for non-adherence. Device required charging every other day and needed to be connected to smartwatch every time shoes were worn. Did not fit optimally in custom-made shoes. Sampling rate not adequate to measure peak pressures.
Lajevardi-Khosh et al., 2019 [51]	Walking boot for lower extremity fracture recovery	Observe patient compliance towards weight-bearing protocols when recovering from lower extremity fractures.	11 patients (5 male and 6 female) aged 19–64 years	Load-monitoring insole (Ambulatory Tibial Load Analysis System) using piezoelectric pressure sensors in silicone gel within silicone elastomer case inserted into base of walking boot. Insole contained three individually cased load sensors—two under the medial and lateral metatarsal heads and one under the heel.	Wear device at all times except sleeping and during ROM exercises.	Average daily weight bearing (from pressure sensors) compared to expected amount of weight bearing to determine compliance. To determine overall compliance, the total number of compliant days was divided by total number of days that the protocol was prescribed for.	Continuous, objective out-of-clinic monitoring for up to 6 weeks on a single set of batteries. Data collected over long periods, so more representative of patient behaviour (patient’s own environment and outside the lab).	Small number of patients and lacking in patient diversification as more weight-bearing protocols exist.

### 3.6. Step Counters

Four studies used step counters to monitor whether a lower limb orthosis was worn [36,40,41,44]. One study used step counters alone to monitor compliance [36] (Table 4), while two studies used step counters alongside temperature sensors [40,41] and one alongside accelerometers [44]. Two studies used step counters that consisted of switches that counted steps when the switch was compressed [36,44], and two studies used an off-the-shelf step activity monitor [40,41]. One study found that the system accurately measured step count and that increasing step count corresponded to wear time [44]. However, another study reported that when the device was used at the participant’s home, readings were inaccurate due to overestimation of the number of steps taken [36]. It was also found that the removal or incorrect positioning of the step activity monitor led to incomplete data and that some users experienced discomfort when wearing the step activity monitor [40]. Additionally, it was mentioned that the tightness or fit of shoe could affect the accuracy of the readings [36]. A reported disadvantage of step counters was that they could not monitor when orthoses were worn during cycling [40] or when standing [41]. Agreement on low orthosis use was seen in both step counter data and patient diaries; however, one study also found that patients were reluctant to provide information [36].

### 3.7. Accelerometers

Six studies utilised three-axis accelerometers to monitor the use of orthoses applied to the hip [33] and lower limb [37,38,42,43,44]. Two studies used accelerometers alone to monitor compliance [37,38] (Table 5), while three studies used accelerometers alongside temperature sensors [33,42,43] and one alongside step counters [44]. Results agreed with patient diaries [37]; thus, accelerometers can accurately measure compliance. On the other hand, when compared to readings from a three-axis accelerometer, it was found that care-giver reported wear times had a −0.55 h bias and the limits of agreement ranged from −2.96 to 1.96 h [37]. It was further reported that accelerometers picked up movement not mentioned in patient diaries, providing an objective insight into orthosis use [37]. In one study, it was reported that accelerometers could be used to accurately measure step count [44]. It was noted that an additional advantage of using accelerometers to monitor compliance was that they reveal the types of activities users perform while wearing their orthotic devices—such as activities that require less locomotion [38]. A disadvantage, however, was that it could be difficult to tell the difference between someone merely holding or travelling with the orthosis and wearing it as prescribed [37]. It was also mentioned that accelerometers are low cost and easy to attach, so there is no need to create or buy new devices and, additionally, the methodology of using accelerometers is easily reproducible [37].

**Table 4:** Details of the studies using step counters alone to monitor compliance, ordered by year published.

Author and Year	Orthosis Prescribed	Study Aim(s)	Population Demographics	Description of Technology	Instructions for Use	Wear Time Estimation	Pros of Method	Cons of Method
Sykes et al., 1996 [36]	Reciprocating gait orthoses (RGO) for spinal cord lesions	Objectively measure the number of steps taken while wearing the orthosis. Objectively measure home use of the orthosis, with and without electrical stimulation.	5 patients aged 24–37 years	Contact switch combined with electronic counter (Syrelec, Farnell, Leeds, UK). Switch consisted of two pieces of aluminium foil (30 mm × 30 mm) separated by plastic sponge foam (4 mm thick). Switch was enclosed in vinyl pocket and attached to heel area of the base of an ankle foot orthosis on the RGO. Switch was connected to a counter attached upright to the thigh area of the RGO. Counter, powered by lithium battery, could display up to 999,999 steps.	Monitoring of RGO use at home for 18 months.	When the contact switch is compressed, one step is registered using the counter. Readings were taken at intervals and the previous recordings were subtracted from the latest recording.	Reliability of step counter was good in laboratory setting. Low use of orthosis was seen in number of steps taken and in patient diaries.	Accuracy decreased when subject started using RGO at home (possibly due to tightness of shoelaces or deterioration of sponge in switch mechanism). Participants were reluctant to provide information.

**Table 5:** Details of the studies using accelerometers alone to monitor compliance, ordered by year published.

Author and Year	Orthosis Prescribed	Study Aim(s)	Population Demographics	Description of Technology	Instructions for Use	Wear Time Estimation	Pros of Method	Cons of Method
Weber et al., 2013 [38]	HEPHAISTOS unloading orthoses for calf muscle unloading	Observe compliance and adherence to the orthoses.	11 healthy male participants aged 20–45 years	Portable three-axis digital accelerometers with replaceable battery (X1-6A, Gulf Coast Data Concepts, Waveland, MS, USA) fixed to shaft of orthosis using Velcro^®^ strips. Set up to automatically start and stop recording data every day (5 a.m.–12 p.m.) on a built-in SD card. New file created every two hours and data retrieved during weekly visit using USB connector.	Wear orthosis during all daily activities for 56 days.	Acceleration analysis using R program. Moving window over 40 samples with no overlap was applied to longitudinal axis each day of each week. For each window, standard deviation (SD) of accelerometer values was calculated. Task-related previously identified SD values (sitting ≈ 0.03 G, standing ≈ 0.01 G, walking ≈ 0.3 G, stair ascending ≈ 0.4 G, and descending ≈ 0.55 G). Activities classified by number of samples ≥ 0.1 G in daytime SD samples compared to total number of SD samples.	Provided control tool. Showed participants wore orthosis during light activities (less locomotive).	No direct proof activity monitor data showed good compliance with protocol.
Griffiths et al., 2022 [37]	Abduction brace for clubfoot	Validate brace wear monitoring method in an infant population.	11 patients aged less than 1 year old	Three-axis accelerometer (activPAL, PAL Technologies, Glasgow, UK) with sampling rate of 20 Hz attached to centre of foot abduction orthoses. Algorithm used summation of accelerometer readings from three axes.	Wear for up to 7 days.	Algorithm used to calculate activity count from raw acceleration to quantify amount of movement. Each time period assigned to wear, and non-wear based on activity count threshold obtained from initial analysis of data from three participants. Algorithm provided wear status every second.	High agreement between results and diary. Showed movement missed by diary. Low cost and easy to attach accelerometers, so no need to create or buy new devices. Method easily reproducible.	Non-wear related movements (e.g., travelling with device that is not worn) could cause errors in wear time estimation. Algorithm needed to be validated against more robust measures. More participants should be recruited.

### 3.8. Combination of Sensors

Seven studies used a combination of sensors [33,39,40,41,42,43,44] (Table 6); three studies used temperature sensors and accelerometers to monitor compliance [33,42,43], two studies used step counters alongside temperature sensors [40,41], one study used temperature and pressure sensors [39], and one study used step counters alongside accelerometers [44]. One study using temperature sensors alongside accelerometers observed a clear overestimation in self-reported compliance [33]. One study found that the mean difference between the adherence monitor and log recordings was 0.4 min [40], while another study, utilising a peak detection algorithm, found the estimated mean wear time to be 207.3 min when the actual mean wear time was 206.9 min [43]. A further study found good levels of agreement between an accelerometer and step counter at observing step count with the mean differences ranging from −1.2 to 2.1% [44]. The use of temperature sensors and step counters was found to help understand factors of adherence and improve treatment [41]. Another study found that a drawback of using temperature and pressure sensors together was that for optimal results, the sensors were required to be still [39]. Limitations with data collection [39], extraction [33], and storage [42] were experienced in some studies; for example, internet connection problems were reported when recording data in one study [39], and another mentioned that in order to download the data collected from the sensors, the orthosis had to be removed [42]. It was also reported that compliance was difficult to measure using temperature sensors alone, so pressure also needed to be measured [39]. 

## 4. Discussion

The results provide an exclusive insight into the techniques that currently exist to monitor orthosis use objectively. More than half of the studies found used temperature sensors to monitor compliance [21,33,34,39,40,41,42,43,45,46,47,48,49,50]. Many relied on a threshold to discern between times when the orthosis was worn and when it was not [21,31,33,37,42,45,46,47,48,50]. Although threshold values were often decided after repeated testing, it is difficult to find a cut-off or threshold temperature that applies to any climate or ambient temperature. To overcome this issue, some studies used two temperature sensors so that a threshold value could be determined using the difference between the temperature readings [34,40,41,50]. Equally important to note is that a few of these temperature sensors needed to be integrated into the orthosis [21,43,46]. Although this approach may reduce the form factor of the device and make it easily attachable to orthotic devices of all sizes, modifying the structure of the orthosis may have an impact on its function. 

Pressure sensors were also used to monitor compliance [31,32,35,39,51,52]. These sensors were used the most in foot insoles [31,32,35,51,52], highlighting their flexibility and compactness. An additional advantage of using pressure sensors is that there is scope to use the sensor beyond estimation of wear time. For example, pressure sensors can be used to determine whether the orthosis is well-fitted or if it is too loose or tight. Furthermore, they also can be used to calculate the magnitude of the force the orthosis applies to musculoskeletal structures. 

Accelerometers were used in studies to monitor movement and activity [33,37,38,42,43] and sometimes to estimate step count [44]. Accelerometers provide clear indications of when there is movement and when there is not. While movement does not directly correspond to wear time, these sensors may be used more easily to investigate the types of activities patients do when wearing their orthosis [38]. 

The final type of technology used to monitor compliance was a step counter [36,40,41,44]; this provided a useful tool for lower limb orthoses. Step counters were used in isolation [36] in only one study, while in the others, a combination of two sensor types was used [40,41,44]. Combining sensor types provides more information, meaning that better estimates could be made about orthosis wear time. It may be possible for users to mislead the estimation of wear time by leaving the orthosis in a warm environment for temperature sensors, moving the device around to simulate movement for accelerometers, or applying extra loading on pressure sensors. However, the use of a combination of sensors will contribute to increasing the accuracy of wear time estimation.

The majority of the studies included in this review examined orthoses for the lower limb [31,32,35,36,37,38,40,41,42,43,44,48,49,50,51,52], highlighting the need for the further development of objective monitoring techniques for the upper limb. This may be because of the need for smaller form factor sensors and associated hardware to avoid interference with upper limb function. All upper limb studies employed temperature sensors [21,34,39,45,46], with one using temperature sensors alongside pressure sensors to monitor compliance [39]. This further highlights the lack of evidence demonstrating the feasibility of a range of objective compliance-monitoring techniques for the upper limbs.

No instances were found in which intelligent material systems or smart materials were employed to create distinct devices or include sensing elements into an orthosis design. In the future, these materials may enable a more seamless incorporation of sensing elements into orthoses.

The challenge of monitoring compliance is not new and is one that should be considered seriously. Without compliance to treatment, the effectiveness of the treatment provided cannot be inferred. The prescription of an orthotic device is usually the consequence of a medical condition diagnosis and, therefore, it is given with the aim to improve the symptoms of the condition, whether that is the correction of a joint deformity, immobilisation, muscle repair, or pain relief. Non-adherence to orthotic treatment could be detrimental in some medical conditions. For example, the onset of conditions such as osteoarthritis is affected by biomechanical loading [53]; therefore, it is important to ensure patients comply and wear their orthoses as recommended to maximise their impact on the affected joint. Thus, the accurate measurement of wear time is essential to provide the best care to patients. Compliance is frequently monitored using patient-reported measures, such as questionnaires, diaries, or upon enquiry during a follow-up appointment, as these are easy to implement and low-cost. However, these measures are subjective and are prone to bias; patients have been found repeatedly to overestimate their levels of compliance [16,21] and can also easily forget when they wore their orthosis when asked about compliance later. From a research perspective, the use of subjective methods when monitoring compliance leads to difficulties when comparing study results, as there may be inconsistencies when comparing results from questionnaires and diaries. In contrast, the use of objective methods allows for easy comparison, whether that is the accuracy of a certain monitoring technique or the wear time estimation of different orthotic interventions.

While objective monitoring reduces bias, it is important to keep the stakeholders in mind. These include patients, their family, caregivers, clinicians, therapists, and sometimes can also include orthoses manufacturers. For example, several studies included in this review investigated the use of orthosis for clubfoot in infants [31,37,49,50]; in this situation, the compliance to the orthoses depends on the parent, guardian, or carer of the infant and not the infant themselves. Therefore, despite the objective monitoring of compliance, there are other influences on compliance that may not be accounted for. Socioeconomic factors, the associated healthcare system, issues arising from the patient’s condition, the prescribed therapy, and the patient’s personal circumstances can all influence compliance to treatment [54]. Objective monitoring methods are worthless if they are impractical, unusable, or uncomfortable. Ideally, the recommended objective monitoring technique would be one that is compact and minimalistic, so that it does not add to any discomfort that may be faced by the patient already wearing an orthotic device. With the increasing use of technology and the introduction of many apps to monitor a wide range of health conditions, the use of objective monitoring in healthcare is not something new. Some patients are comfortable with the idea of monitoring through mobile phones and are also confident that their privacy will be protected [55]. However, it is equally important to note that some patients feel that telemonitoring is intrusive [56]. This feeling may negatively affect compliance to the orthotic treatment. It is also important to keep in mind the relationship between a patient and their clinician [17]; it could easily be inferred by the patient that by using objective compliance-monitoring methods, the clinician does not trust them to wear their orthosis as prescribed, or does not wish to follow-up with them in person, and therefore, it is imperative that compliance-monitoring techniques are as beneficial to patients as they are to clinicians and researchers. 

Many studies included in this review used technologies that are best suited to a laboratory environment, as they had a large form factor, had visible wires, or needed to be removed to obtain the data from the device. With many patients citing a lack of aesthetic value of orthoses [15,17,57], the addition of a compliance-monitoring device that increases the visibility or form factor of the orthosis would likely negatively impact compliance further. One way to make wearing an orthosis and a monitoring device less daunting could be the introduction of an app that works alongside an objective monitoring technique to actively display wear time statistics to the user. This allows them to be more involved in their care. For clinicians and therapists, this may provide insights into patient behaviour, and they can use the information obtained to provide the best care for their patients. This could include the change of orthoses if one type is not working well or the consideration of alternative treatment options. 

When designing an objective method to monitor compliance, data sample rate is important. Some of the included studies used a variety of sampling rates, depending on the length of the study. Battery life is also important as this dictates the longevity of the device; studies used both replaceable and rechargeable batteries. Returning to the key stakeholder, the user’s opinions need to be considered when implementing these approaches, as charging and replacing the battery of the device is further burden on top of remembering to wear an orthosis and may be physically challenging for some patients.

Recommendations can be made for future studies that aim to design or implement objective monitoring techniques for orthosis use. Devices monitoring compliance need to be reliable and accurate. Out of the included studies, temperature sensors have frequently been reported to accurately measure compliance. When investigating the accuracy of objective monitoring techniques consisting of sensors, it is imperative that, where possible, the ground truth to which the sensor system accuracy is being compared to is also objective. User-reported orthosis wear time is subjective and prone to bias; therefore, subjective measures will not be fully representative of the ground truth. 

Cost and form factor are also important—if the device is low-cost and small, it is more likely to be applicable to a wide range of orthoses and usable for different anatomical locations. Furthermore, if a device is low-cost and user-friendly, it increases the practicality of the technology and its applicability in clinical settings with less resources. Consequently, it is no surprise that temperature sensors are the most common sensor type when monitoring compliance; their low cost and compactness allow for them to be used in upper limb, hip, and lower limb orthoses. Moreover, the high number of studies utilising temperature sensors to successfully monitor compliance instils confidence for future studies looking to employ this method to monitor orthosis use. 

However, where there are possibilities of high temperatures being reached, such as due to being placed under a foot experiencing high pressures or in a geographical region with a warmer climate, temperature sensors should be used with caution, or an alternative type of sensor should be used, as accuracy could be compromised. While temperature sensors have been shown to objectively monitor compliance, if information about orthosis fit or the user’s levels of activity is required, other sensors should be sought. Pressure sensors, more commonly used in lower limb orthoses, can monitor compliance while having the potential to provide additional information regarding the pressure at the orthosis–skin interface, the forces applied by the orthosis, or how well the orthosis is fitted by either the orthotist or user themselves. When monitoring compliance alone, pressure sensors could be favourable to temperature sensors, as it is relatively simpler to find a threshold value to distinguish between donning and doffing and extreme temperatures are not of concern. 

Accelerometers and step counters have proven to be valuable in studies looking into the movement and ambulatory activity of orthosis users. While step counters are useful in monitoring the use of lower limb orthoses, accelerometers, provided they are compact, can be applied to upper limb and hip orthoses to monitor how active patients are while wearing their orthosis. That said, accelerometers should be used with caution when measuring compliance, as it is easy for users to imitate wear time by simply moving the instrumented orthosis. 

Ultimately, sensor systems should be chosen based on their application and on study-specific parameters, as all sensor types have their own advantages and disadvantages. If orthosis fit is being investigated, pressure sensors are well suited as they can provide information regarding the forces applied by the orthosis at the orthosis–skin interface, which can be correlated to how well the orthosis is fitted. If the main interest lies in improving the efficacy of orthosis treatment, it may be valuable to utilise accelerometers as they offer an insight into the mobility and activity levels of orthosis users while they wear their orthosis. Furthermore, where the forces applied by the orthosis to the skin are fundamental to its function, pressure sensors can be used to monitor compliance and orthosis efficacy. Temperature sensors can also be used to monitor efficacy as high temperatures could correlate to high pressures. Finally, with the intention of monitoring and bettering compliance, temperature sensors, accelerometers, and pressure sensors have all been shown to provide useful insights, and therefore, sensors can be chosen based on parameters such as the desired sampling rate, ambient temperature, the purpose of the orthosis, anatomical location, and data-processing techniques. 

Using a combination of sensors is recommended where a multi-faceted picture of orthosis use is desired, such as studies of both compliance and activity, or when examining pressure applied to a specific anatomical region. A combination of sensors could also be used to obtain more reliable and accurate measurements of compliance alone than that which could be quantified with a single sensor, with wear time being measured based on multiple data types. 

Patient and public involvement during the development of the device is invaluable, as this offers insight into the reasons for non-compliance and provides user perspectives on objective monitoring devices [16]. Patients can provide suggestions and feedback on the appearance, comfort, and usability of the device. Clinicians’ insight can also provide useful information regarding treatment duration, regularity of check-ups, and orthosis types, as these factors can determine aspects of the compliance monitoring device; for instance, required sample rate, means of data extraction, and practicalities of data storage. All these inputs are beneficial, especially in the testing of novel orthotic treatments, as this can lead to the appropriate modifications being made. To summarise, as has been previously reported, the ideal compliance-monitoring device would be unobtrusive to minimise discomfort and maximise patient cooperation, objective to provide accurate and reliable data, practical to maximise portability and minimise cost, reasonably priced, sensitive, and user-friendly [58,59,60].

There is a clear need for objective techniques to monitor compliance, but this does not mean that patient-reported outcomes are not valuable—in fact, it is the opposite. Patient-reported outcome measures may be subjective and prone to bias when monitoring compliance, but they provide useful information with regards to reasons for non-compliance and general feedback about a device. Sensor-based methods to monitor compliance are able to do what would otherwise be time-consuming and prone to errors if carried out by a human. Ideally, when investigating compliance, both sensor-based and patient-reported measures would be used, as the former provides real-time, accurate data about whether the patient is wearing the device, and the latter provides patient insights while using the device and any reasons for non-compliance. 

While care was taken in developing a search strategy that included a range of keywords synonymous with the three main concepts: orthoses, compliance, and objective, it may have been possible that some studies were not retrieved from the search as they used a different term for compliance or adherence, or used another term for orthoses, for example. However, the terms used in the strategy were obtained by reading the relevant literature, and so it is difficult to predict the usage of and include terms that have not occurred elsewhere. Another point to note is that the search was conducted on five databases, and although the databases chosen covered a wide range of resources, there is a possibility that other databases could have relevant records that were not included in this review. Nevertheless, this review retrieved records from a variety of sources using a thorough search strategy encompassing multiple terms for each main concept and has identified objective compliance-monitoring techniques that can be utilised and enhanced by both researchers and clinicians in future work.

## 5. Conclusions

Objective, sensor-based methods to monitor use of orthoses for the extremities have been employed in a variety of ways. The methods include the use of temperature sensors, accelerometers, pressure sensors, and step counters to monitor orthosis wear time accurately. All sensor types have their own advantages and disadvantages and, therefore, future studies should select sensors and sample rates based on study-specific parameters, as there is no one sensor that suits all scenarios. Objective monitoring provides invaluable data that are beneficial in both clinical and research settings. In clinical settings, it can be used to monitor treatment progress and to see whether the current orthosis is working or if alternative options, such as a different orthosis or surgery, are better. Objective monitoring is also a powerful tool in research as it can be utilised to assess and validate the effectiveness of both existing and novel orthotic interventions. The ideal solution to monitoring compliance would consist of both sensor-based and user-reported aspects that, in combination, provide an all-encompassing picture of the status of treatment. Objective methods are essential in providing true wear time data to know if, and when, patients are wearing their orthosis, and if patient-reported measures are necessary to understand the factors that influence compliance, including why one may or may not wear their orthosis as prescribed.

## Figures and Tables

**Table 1:** A summary of the study characteristics of the included studies.

Study Characteristic		Number of Studies
Design	Randomised controlled trial	3
	Observational	20
Country	United Kingdom	5
	Netherlands	3
	Switzerland	2
	Germany	3
	Portugal	1
	United States of America	7
	China	1
	Australia	1
Sample	Patient	15
	Healthy	7
	Patient and healthy	1
Sample size	Range	2–124
Age (years)	Range	0.25–86
Orthosis application region	Upper limb	5
	Hip	2
	Lower limb	16
Compliance-monitoring method	Temperature sensors	8
	Pressure sensors	5
	Step counters	1
	Accelerometers	2
	Temperature sensors and accelerometers	3
	Temperature and pressure sensors	1
	Temperature sensors and step counters	2
	Accelerometers and step counters	1
Medical condition orthosisis used for	Diabetes	6
	Clubfoot	4
	Postoperative (shoulder surgery)	3
	Carpel tunnel syndrome	1
	Hip fracture prevention	1
	Hip dysplasia	1
	Lower limb fracture recovery	2
	Foot drop	1
	Spinal cord injury	1
	No specific condition	3

**Table 6:** Details of the studies using a combination of sensors to monitor compliance, ordered by year published.

Author and Year	Orthosis Prescribed	Study Aim(s)	Population Demographics	Description of Technology	Instructions for Use	Wear Time Estimation	Pros of Method	Cons of Method
Bus et al., 2012 [40]	Footwear for diabetes	Assess validity and feasibility of temperature-based adherence monitor to monitor footwear use.	11 healthy participants (8 male, 3 female, mean age 42.0 ± 9.4 years) and 14 patients (11 male, 3 female, mean age 56.2 ± 12.9 years)	Temperature-based adherence monitor (@monitor) measuring 35 × 15 × 5 mm consisting of two temperature sensors, battery, and data logger. Temperature collected every minute (maximum sampling rate) at the inner lateral shoe border. Sensors placed in a foam pad and affixed using tape. Number of steps taken recorded using step activity monitor (StepWatch, Orthocare Innovations, Edmonds, WA, USA) attached to the ankle.	Healthy: Don/doff several times while wearing monitor for one day in climate-controlled hospital setting. Subset of healthy participants instructed to wear device between 4 and 7 days while recording donning/doffing times. Patients: tested in own prescription footwear for 7-day period with step activity monitor. Instructed to wear at all times apart from when taking a shower/bath.	Average temperature difference between both temperature sensors was calculated in all samples that showed a difference greater than ≥0.3 °C. When the difference between the two sensors in sample was >25% of average, the shoes were classified as being worn and not worn when <25%. Number of steps taken and the time shoes were worn was calculated for each day. Adherence calculated as percentage of daily steps taken whilst wearing footwear and averaged over number of days of data collection.	Valid and feasible adherence data obtained from temperature sensors. Two sensors reading temperature could contribute to higher accuracy and improve sensitivity to temperature change. Small and light device. Performance of temperature sensors checked during the summer and autumn and no difference was found.	Some overestimations of donning and doffing times when data obtained over multiple days. Few issues with usability. Experienced loss of data due to an error on temperature-monitoring device. Removal or incorrect placement of step activity monitor by patient could cause incomplete data. Cycling activity could not be distinguished from ambulatory activity. Discomfort experienced when wearing step activity monitor. System performance in extreme temperatures unknown. Only sampled once every minute so donning or doffing within 1 min period may not be registered. Not tested in orthoses. May not be enough space to fit monitor in a total contact device.
Waaijman et al., 2013 [41]	Custom-made footwear for diabetes	Objectively assess adherence to prescribed footwear during ambulation in diabetic patients at high risk for ulceration. Evaluate determinants of adherence in patients.	107 patients (93 male and 14 female) aged 54–73 years	Temperature-based adherence monitor (@monitor) measures 35 × 15 × 5 mm and consists of two temperature sensors, battery, and data logger. Temperature collected every minute (maximum sampling rate) at the inner lateral shoe border below the ankle. Number of steps taken recorded every minute using step activity monitor (StepWatch, Orthocare Innovations, Edmonds, USA) strapped to the ankle.	Footwear use for 7 consecutive days. Patients asked to step activity monitor at all times apart from when taking a shower or bath or when facing discomfort.	Adherence calculated as total number of steps whilst wearing the prescribed footwear divided by total number of steps. When step activity was recorded but @monitor did not record use, it was assumed patient was walking barefoot or in non-prescribed footwear. Time away from home was reported in daily log and used to classify wear time data for periods at home and away from home.	Objective data collected on adherence. Helped to understand predictive factors of adherence and improve treatment.	Adherence was not measured when standing. Still dependent on daily logs to determine wear time (subjective). Methods needed to ensure patients do not take off step activity monitor.
Telfer et al., 2014 [42]	Footwear for diabetes	Assess feasibility of foot orthoses with embedded temperature sensors for monitoring foot temperatures. Determine if temperature measurements could be used to detect periods of activity.	10 healthy participants aged 22–46 years	Temperature sensor (iButton DS1920Z, Maxim Integrated Products, San Jose, USA) with ability to store 2048 individual measurements and resolution of ±0.125 °C enclosed in stainless steel shell (16.25 mm diameter, 5.95 mm thick) positioned distal to medial arch in contact with foot under first and second metatarsal heads. Data collected at 3 min intervals to allow for 4 days of temperature measurements. Activity monitor (ActivPAL, PAL Technologies, Glasgow, UK) worn on thigh to give estimate on energy expenditure based on steps taken and posture.	Wear for 4 days.	Algorithm developed to predict high activity from temperature data using the rate of change of the difference between plantar surface and ambient temperatures. Optimum threshold rate of change, averaging of temperature data, and offset of temperature data in relation to activity data (to account for delays in response of foot temperatures to activity) used to optimise performance of algorithm.	Demonstrated feasibility. System could be used to measure compliance with foot orthosis interventions. Potential to increase understanding of how activity affects plantar and in-shoe temperatures.	Limitations with respects to data storage and resolution. Low sampling rate to measure temperatures for several days. Orthosis needed to be removed to download data from sensors.
Miller Renfrew et al., 2020 [44]	Ankle foot orthoses for foot drop	Assess validity and level of agreement between PALite and Odstock Dropped Foot Stimulator (ODFS) Pace activity in measuring step count.	16 healthy participants (9 male and 7 female) aged 18–65 years	Accelerometer (PALite, PAL Technologies, Ltd., Glasgow, UK) placed on lateral aspect of lower leg with waterproof dressing. Step counter (ODFS Pace activity logger, OML) was worn on waistband (no stimulation delivered), and steps were counted using a pressure sensitive switch taped to heel of insole inserted into participant’s shoe.	Walk for 5 min at 3 speeds: normal (1.3 ms^−1^), slow (0.4 ms^−1^), and fast (1.7–2.0 ms^−1^).	PALite uses accelerometer values to detect movement of the leg shank during the step cycle. ODFS Pace activity logger records heel contacts on the pressure switch during heel strike.	PALite measured step count more accurately than ODFS. Validity for both in measuring step count. PALite could be used to monitor adherence with other orthotic devices.	Devices agreed less at normal and slow walking speeds than at fast walking speed.
Menz & Bonanno 2021 [43]	Foot orthoses for lower limb disorders	Validate a temperature sensor for monitoring adherence to foot orthoses	10 healthy participants (7 male and 3 female) aged 38–50 years	Miniature (9 × 13 × 4.5 mm) temperature sensor (Orthotimer, Rollerwerk Medical Engineering, Balingen, Germany) embedded into proximal region of medial longitudinal arch. Wearable accelerometer (Fitbit Zip, Fitbit Inc., San Francisco, CA, USA) attached to the exterior of right shoe. USB temperature data logger (Instrument Choice, Adelaide, Australia) recorded ambient temperature. Smartphone application (HoursTracker, Cribasoft, Round Rock, TX, USA) used to record the time the orthoses were placed in the shoes and when they were removed. Temperature recorded at one-minute intervals and physical activity every 15 min.	Wear orthosis for between one and seven hours per day over a five-day period. Then remove orthoses while keeping shoes on. Wear time for each day was randomised for each participant. Instructed to always keep USB ambient temperature data logger near them.	Nine algorithms were evaluated. Four used absolute cut-off temperature values from the sensor, four used temperature values from the sensor relative to ambient temperature. The ninth algorithm identified the largest one-minute increases and decreases in temperature from the sensor corresponding to donning and doffing.	Provided valid orthosis wear time data. Ambient temperature found not to affect temperature sensor readings.	Sensor accuracy tested in only one location. Testing conducted in largely sedentary conditions rather than physically active populations. Sensor embedded in orthosis and not in shoe, so identification of shoe removal vs. orthosis removal may differ. Further evaluation needed to determine accuracy at lower sampling rates.
Wong et al., 2022 [33]	Hip protector for hip fracture prevention	Quantify compliance with hip protector use in an elderly population. Investigate effects of different hip protector holders on compliance.	13 patients aged 76–86 years	Temperature sensor (to differentiate between the ambient and body temperatures), three-axis accelerometer (to detect falls), flash memory (256,000-sample storage capacity), battery, and backup battery. For each data point, the date, time, temperature, and acceleration in each axis were recorded. Data were sampled every 30 s and were transferred wirelessly to a computer.	24 h for 4 weeks (except when bathing).	Wear time was assessed using both temperature sensor and accelerometer. If the temperature was higher than the personalised threshold (determined as 1 °C less than temperature recorded after an individual wore the hip protector for 15 min), and if changes in the acceleration between the previous two points were higher than the threshold value of 0.023 g, the hip protector was considered to be worn.	Demonstrated feasibility of compliance quantification. Clear to see overestimation in self-reported compliance. Provided understanding of actual usage and efficacy.	Some devices failed to extract data, meaning a potential risk in damage to the monitor.
Dinis et al., 2023 [39]	Upper limb orthoses for carpal tunnel syndrome	Investigate device that monitors orthosis use, tightness, and potential inflammation reaction.	2 healthy participants	Arduino ^®^ UNO R3 connected to three resistive force (pressure) sensors (0.3 mm thickness) and three temperature sensors. Data transfer performed through Bluetooth and data shown in Android application while saved in database in real-time.	Wear for 2 consecutive days.	Temperature and pressure increase when orthosis worn and decrease when unused.	Objectively measured compliance. Pressure and temperature sensors were low cost.	Sensors needed to be kept still for optimal results. Internet connection problems when recording data. Compliance difficult to measure with temperature sensor alone, so pressure also needed to be measured.

## Data Availability

Not applicable.
